# Adaptive Traits to Improve Durum Wheat Yield in Drought and Crown Rot Environments

**DOI:** 10.3390/ijms21155260

**Published:** 2020-07-24

**Authors:** Samir Alahmad, Yichen Kang, Eric Dinglasan, Elisabetta Mazzucotelli, Kai P. Voss-Fels, Jason A. Able, Jack Christopher, Filippo M. Bassi, Lee T. Hickey

**Affiliations:** 1 Centre for Crop Science, The University of Queensland, Queensland Alliance for Agriculture and Food Innovation, Brisbane, QLD 4072, Australia; yichen.kang@uq.net.au (Y.K.); e.dinglasan@uq.edu.au (E.D.); k.vossfels@uq.edu.au (K.P.V.-F.); 2 Council for Agricultural Research and Economics (CREA)—Research Centre for Genomics and Bioinformatics, 29017 Fiorenzuola d’Arda (PC), Italy; elisabetta.mazzucotelli@crea.gov.it; 3 School of Agriculture, Food and Wine, Waite Research Institute, The University of Adelaide, Urrbrae, SA 5064, Australia; jason.able@adelaide.edu.au; 4 Centre for Crop Science, The University of Queensland, Queensland Alliance for Agriculture and Food Innovation, Leslie Research Facility, Toowoomba, QLD 4350, Australia; j.christopher@uq.edu.au; 5 International Center for the Agricultural Research in the Dry Areas, Rabat 10000, Morocco; F.Bassi@cgiar.org

**Keywords:** drought adaptation, fusarium, stay-green, root architecture, association mapping, water use

## Abstract

Durum wheat (*Triticum turgidum* L. ssp. *durum*) production can experience significant yield losses due to crown rot (CR) disease. Losses are usually exacerbated when disease infection coincides with terminal drought. Durum wheat is very susceptible to CR, and resistant germplasm is not currently available in elite breeding pools. We hypothesize that deploying physiological traits for drought adaptation, such as optimal root system architecture to reduce water stress, might minimize losses due to CR infection. This study evaluated a subset of lines from a nested association mapping population for stay-green traits, CR incidence and yield in field experiments as well as root traits under controlled conditions. Weekly measurements of normalized difference vegetative index (NDVI) in the field were used to model canopy senescence and to determine stay-green traits for each genotype. Genome-wide association studies using DArTseq molecular markers identified quantitative trait loci (QTLs) on chromosome 6B (*qCR-6B*) associated with CR tolerance and stay-green. We explored the value of *qCR-6B* and a major QTL for root angle QTL *qSRA-6A* using yield datasets from six rainfed environments, including two environments with high CR disease pressure. In the absence of CR, the favorable allele for *qSRA-6A* provided an average yield advantage of 0.57 t·ha^−1^, whereas in the presence of CR, the combination of favorable alleles for both *qSRA-6A* and *qCR-6B* resulted in a yield advantage of 0.90 t·ha^−1^. Results of this study highlight the value of combining above- and belowground physiological traits to enhance yield potential. We anticipate that these insights will assist breeders to design improved durum varieties that mitigate production losses due to water deficit and CR.

## 1. Introduction

**Figure 1:**
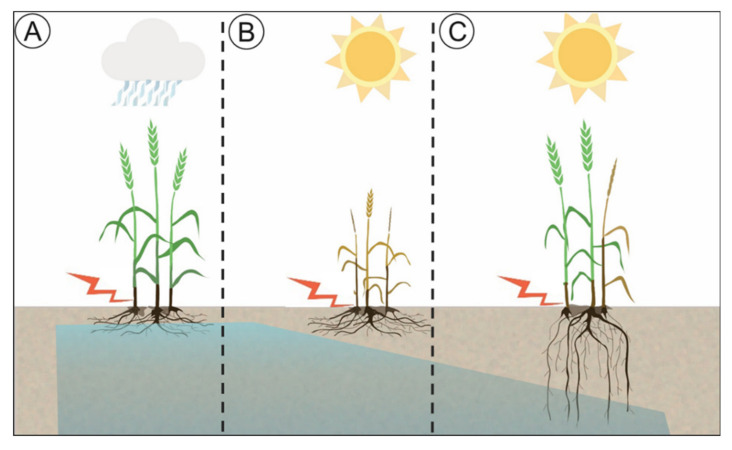
Illustration displaying the effect of crown rot infection on yield under different scenarios: (**A**) Minimum yield losses when water is available through the growing season, i.e., intermittent rainfall events. (**B**) Maximum yield losses when water is limited, and the root system architecture is not designed to reach moisture at depth. (**C**) Less severe yield losses achieved under water-limited conditions when there are optimised below and aboveground trait combinations (i.e., root system architecture and stay-green).

Durum is typically grown under rainfed conditions in the semiarid regions of the world [1]. Therefore, yield is strongly influenced by the quantity and timing of rainfall throughout the growing season. Increased variability in rainfall is predicted for most durum growing regions worldwide, particularly the Mediterranean region, suggesting that drought will continue to have an impact on production into the future [2]. Durum wheat is also very susceptible to crown rot (CR), a stubble-borne disease, especially when high inoculum levels coincide with terminal drought (Figure 1B). CR continues to be a destructive disease globally and has been particularly damaging due to dramatic changes in cropping systems, including intensive cropping, the introduction of minimum tillage practices and a rapidly changing climate [3,4]. Among the small grain temperate cereals, durum wheat is the most susceptible and, therefore, can experience greater yield losses due to CR infection compared to bread wheat and barley [5]. In Australia, several *Fusarium* species associated with CR have been detected in cereal hosts [6], including *Fusarium pseudograminearum*, which is common throughout eastern Australia, and *Fusarium culmorum*, which is less common but frequent in high-rainfall regions in Victoria and South Australia. The pathogen is also prevalent in other growing regions of the world affecting production of winter cereals in the Pacific Northwest of the USA [7,8], Italy, North Africa, and The Middle East [9,10] and recently in China [11].

Symptoms of CR include whiteheads, where spikes prematurely die in response to infection. This is due to mycelium colonization of the roots and base of the plant, including stems, which restricts translocation of water [12]. The symptoms are exacerbated under water-deficit conditions during flowering time and the grain-filling period [13]. This can result in highly shriveled grain and reduced seed quality [5,14,15,16,17,18]. The downgrade in quality due to decreased test weight leads to a significant reduction of the farmers’ potential income [19]. While the mechanisms underlying the variation in CR severity displayed in drought-affected environments remain unclear, water stress has also been reported to enhance CR proliferation and to spread under glasshouse conditions [20].

Although CR is a chronic problem throughout the Australian wheatbelt, losses could be minimized if crops have sufficient water availability (Figure 1A) or have access to water that is stored deep in the soil profile (Figure 1C). CR has been one of the factors limiting the expansion of durum production in Australia despite the high demand for Australian durum grain from European markets. Currently, all durum varieties are very susceptible to CR; for this reason, the genetic improvement of CR tolerance in durum wheat germplasm is still in its infancy. To date, the main focus for decreasing the impact of CR on yield has been on inoculum reduction through management practices [21,22,23,24,25,26]. An alternative or complementary strategy could involve breeding durum varieties equipped with physiological traits that improve the balance between water supply and demand to ultimately increase yield stability under CR conditions [13].

A number of physiological traits could be exploited to reduce water stress and to maximize the length of the grain-filling period. For instance, early flowering as an avoidance mechanism can reduce the impact of terminal drought and heat stress during grain filling [27,28], which may also limit the impact of CR. In part, this phenomenon of earlier maturity explains the smaller yield loss typically displayed by barley crops compared to common wheat [8]. Moreover, crop varieties with “stay-green” exhibit delayed canopy senescence and can result in higher yield under water-deficit conditions, as reported for bread wheat [29,30], barley [31], sorghum [32,33], and maize [34]. Stay-green can be achieved by modulating either canopy development or root architecture. For instance, key loci underpinning stay-green in sorghum influence root architecture and serve to increase water extraction and to improve drought adaptation [35]. Therefore, conserving water during vegetative growth and improving access to stored soil moisture through optimized root systems [36] could maximize the grain-filling period for durum crops under drought and CR conditions.

A number of quantitative trait loci (QTLs) for root traits have been reported in durum wheat [37,38], including the major QTL for seminal root angle (SRA) *qSRA-6A* [39]. The study of *qSRA-6A* identified two main haplotypes: hap1 (associated with narrow SRA) and hap2 (associated with wide SRA), which were found to be independent of root biomass. This highlights the opportunity to manipulate SRA without modifying root biomass and to develop varieties with a range of different root systems [40]. However, the value of *qSRA-6A* to improve yield performance under water-deficit conditions is yet to be determined.

In this study, a subset of durum wheat nested association mapping population (NAM) was evaluated for yield in six rainfed environments, including two environments with high CR disease pressure. Field-based phenotyping and genome-wide association studies (GWAS) were performed to identify QTL associated with CR tolerance and adaptive aboveground traits, including flowering time and stay-green. Here, we investigate the value of these QTL regions in combination with the major QTL for root angle *qSRA-6A* to improve durum yield under drought and CR conditions.

## 2. Results

### 2.1. Variation in CR Severity

In the 2017 yield trial conducted in the CR screening nursery, the panel displayed a wide range of disease responses. The percentage of whiteheads ranged from 1.1–65.2% for the first reading (WH1) and 1.9–77.2% for the second reading (WH2) (Table 1). The mean whitehead rating was higher for the second reading (21.3% versus 37.1%) because plants expressed more symptoms as the disease developed throughout the season. A higher degree of variation in whitehead ratings was evident for the first reading (CV = 65.4%) compared to the second reading (CV = 48.0%). This might suggest that the panel was segregating for a larger number of adaptation traits or mechanisms contributing to CR tolerance early in the season. In the nursery, disease developed evenly across the experiment, and this resulted in moderate broad-sense heritability measures for both WH1 (H^2^ = 0.45) and WH2 (H^2^ = 0.67). The stem browning discoloration scores ranged from 2.3–8.8, and measures between plots were more variable, reflected by a low broad-sense heritability (H^2^ = 0.24). Overall, less phenotypic variation for stem browning was observed in the panel (CV = 19.2%) compared to whitehead ratings (Table 1).

### 2.2. Yield and Component Traits in the Presence and Absence of CR

In 2017, yield trials were conducted in the presence and absence of CR at Warwick, Queensland. In both experiments, variation was observed for yield and component traits including plant height (PH), days to flowering (DTF), thousand kernel weight (TKW) and stay-green traits (Table 1).

Overall, the CV for time to flowering was low in the presence and absence of CR (CV = 2.0%, 2.2%; respectively). In general, variation in stay-green traits and yield was higher in the presence of CR (CV = 11.0–27.0%) compared to the absence of CR (CV = 8.3–13.4%). The additional trait variation observed under CR conditions might be due to additional adaptive mechanisms and/or loci that contribute to trait expression in environments with combined stresses (i.e., water stress and disease pressure). Generally, moderate to high broad-sense heritabilities were observed, except for PH and DTF which were considerably lower in the presence of CR (H^2^ = 0.45 and 0.18, respectively).

Yield in the presence of CR ranged from 0.58–3.94 t·ha^−1^ with a mean value of 2.30 t·ha^−1^, which was less than half of the yield in the absence of CR at Warwick (4.9 t·ha^−1^, Table 1). Furthermore, the CR disease pressure likely inhibited grain-filling and reduced thousand kernel weight (TKW) with a mean value of 37.70 g in the presence of CR in comparison to 41.90 g in the absence of CR. Plant height mean values showed little difference between experiments (i.e., 63.40 cm in the presence and 66.70 cm in the absence of CR). In the presence of CR, the panel of lines displayed a wider range for most stay-green traits (Table 1). Overall, the coincidence of high disease pressure and water stress in the CR screening nursery resulted in faster senescence. For instance, the average number of days from flowering until 50% senescence (MidS) was 16.8 d in the CR screening nursery compared to 25.3 d in the absence of CR. Similar results were observed for the number of days from flowering time to 90% senescence (EndS), with 30.0 d in the presence of CR and 36.3 d in the absence of CR (Table 1).

**Figure 2:**
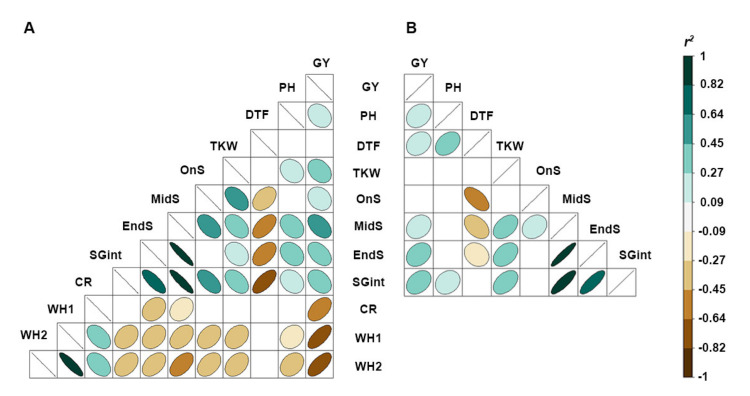
The Pearson’s correlation matrix displays (**A**) stay-green trait correlations with yield in the presence of and (**B**) absence of crown rot in 2017 Warwick, Queensland. The colour gradient of ellipses indicates positive correlations (green) and negative correlations (brown) while the absence of an ellipse indicates that the correlation was not significant (*p* ≥ 0.05). Traits include yield (GY) in tonnes per hectare; PH, plant height; DTF, number of days to flowering; TKW, thousand kernel weight; OnS, number of days from flowering until 10% senescence; MidS, number of days from flowering time until 50% senescence; EndS, number of days from flowering time until 90% senescence; and SGint, area under the curve modelled senescence curve from flowering time to complete senescence.

Stay-green traits (MidS, EndS and SGint) phenotyped in the presence of CR were highly correlated with yield (Figure 2A). In comparison, these traits were only moderately correlated with yield in the absence of CR (Figure 2B). Importantly, a longer grain-filling period supported higher yield performance as the stay-green traits showed positive correlations with TKW, both in the presence and absence of CR. As expected, a strong negative correlation between CR severity scores (CR, WH1 and WH2) and yield was observed in the CR screening nursery.

### 2.3. Association between qSRA-6A and Field Performance

**Figure 3:**
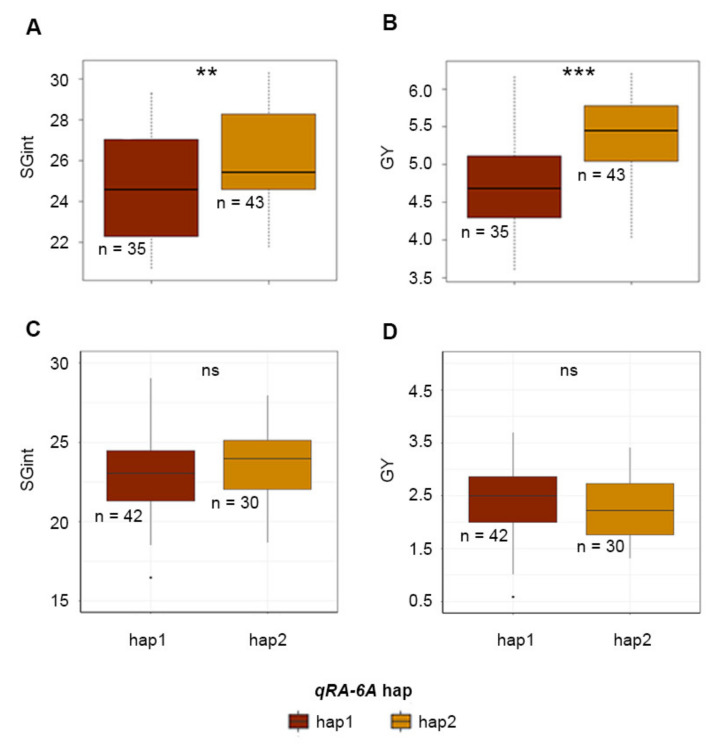
Haplotype effects of seminal root angle quantitative trait loci (QTL) *qSRA-6A* on (**A**) stay-green (SGint) and (**B**) yield (GY) in the absence of crown rot (CR): Haplotype effects of *qSRA-6A* on (**C**) SGint and (**D**) GY in the presence of CR. Significance levels for comparisons of two major haplotypes are indicated at the levels *p* ≤ 0.001 (***) and *p* ≤ 0.01 (**). No significant difference between *qSRA-6A-hap1* and *qSRA-6A-hap2* was noted for SGint as well as GY in the presence of CR. In the boxplots, the line is the median, the box are the bounds for the lower and upper quartile values Q1 = 25% and Q3 = 75 respectively, while the lines below and above indicate the extreme values; values outside the lines are outliers.

The most frequent haplotype groups of the previously reported root angle QTL *qSRA-6A* (hap1 and hap2; Alahmad et al. [39]) were investigated for their effect on field performance in the presence and absence of CR using datasets from Warwick 2017. In the absence of CR, lines carrying *qSRA-6A*-hap1 showed significantly reduced stay-green (SGint) compared to those carrying *qSRA-6A*-hap2 (*p* < 0.01) and significantly higher yield (*p* < 0.001; Figure 3A). Lines carrying the favorable allele displayed a yield advantage of 0.57 t·ha^−1^ (Figure 3B). However, in the presence of CR, no significant differences were observed (Figure 3C,D).

### 2.4. Association between qCR-6B and Field Performance

**Figure 4:**
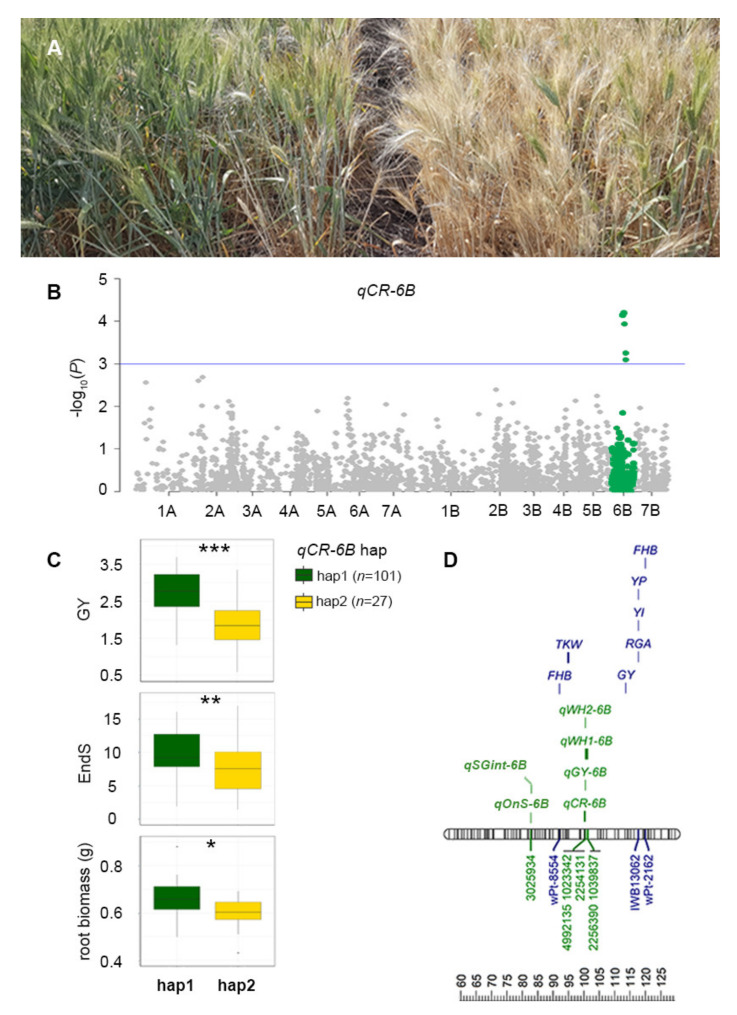
The panel illustrates (**A**) crown rot (CR) severity during the grain filling stage with 5% (left) and 95% whiteheads (right). (**B**) The Manhattan plot shows significant marker-trait association (green) at an arbitrary threshold: −log10(*P*) ≥ 3 (blue horizontal line). The x-axis displays the DArTseq markers on 14 chromosomes; the y-axis is −log10(*P*). The local linkage disequilibrium (LD) block for 5 significant markers representing the CR QTL “*qCR-6AB*” was used for constructing the haplotype network. A total of 26 haplotype variants of the *qCR-6B* for 168 genotypes was observed, and the two major haplotype groups were used for investigating root biomass, stay-green and yield performance under different environments. (**C**) The significant differences between the hap1 variant (favorable allele in green) and hap2 variant (unfavorable allele in yellow) of *qCR-6B* is presented in boxplots. For haplotype trait comparisons, the significance level is indicated as *** (*p* < 0.001), ** (*p* < 0.01) and * (*p* < 0.05). (**D**) A section of chromosome 6B (60–125 cm) displaying the location of QTL was identified in this study along with a previously reported QTL positioned on the Svevo durum physical map. Genomic regions controlling CR severity and symptoms (whiteheads), stay-green and yield from this study (green) were aligned with previously reported QTL associated with traits such as root growth angle, Fusarium head blight (FHB), grain quality and yield (blue).

CR severity index phenotypes for the panel of 168 lines were used for GWAS (Figure 4A). A Manhattan plot describing the association between highly significant markers and CR severity response under CR conditions is presented in Figure 4B. A total of five significant markers were detected on chromosome 6B based on the arbitrary threshold: −log_10_(*P*) ≥ 3. A single QTL (*qCR-6B*) was assigned based on a high level of linkage disequilibrium (LD, *r^2^* = 0.71) between the markers 1023342 (42.43 cm DArTseq V4 consensus map) and 1039837 (53.61 cm DArTseq V4 consensus map) (Figure 4B). Using the five significant markers and their allelic variation in the panel of 168 genotypes, 26 haplotype groups were identified. The most frequent variants were hap1 and hap2 (101 individuals versus 27 individuals; frequency = 60.12% and 16.07%, respectively). Lines carrying hap1 displayed a higher CR severity index compared to those carrying hap2 (6.60 and 5.10, respectively; *p* ≤ 0.001).

The haplotype effect of the *qCR-6B* QTL was investigated for yield performance and stay-green in the presence of CR. Lines carrying hap1 for *qCR-6B* QTL outperformed lines carrying hap2 (2.70 t·ha^−1^ versus 1.91 t·ha^−1^), representing an average yield benefit of 0.79 t·ha^−1^ (*p* ≤ 0.001). In addition, the number of days from flowering until 90% senescence (EndS) was also significantly different between hap1 and hap2 for the *qCR-6B* QTL, with lines grouped to hap1 displaying a longer grain-filling period by an extra 4.24 d compared to those carrying hap2 (*p* ≤ 0.05). Interestingly, while exploring trait relationships, we discovered that lines carrying different haplotypes for *qCR-6B* also showed significant differences in root biomass measured under controlled conditions. Lines with hap1 displayed significantly higher root biomass (0.66 g versus 0.59 g, *p* ≤ 0.05; Figure 4C).

### 2.5. Alignment of QTL Regions Influencing Crown Rot Severity, Water-Use Traits, and Yield

GWAS identified a genomic “hot-spot” on chromosome 6B harboring several key QTLs associated with stay-green traits, CR tolerance and yield. Therefore, results in this study focus on describing trait associations in this region. This included *qPH-6B* for plant height, *qSG-0.1-6B* and *qSG-integral-6B* for stay-green traits, and *qGY-6B* for yield. Interestingly, GWAS using scores for the percentage of whiteheads (WH1 and WH2) in the CR nursery identified QTLs (*qWH1-6B* and *qWH2-6B*) that represented the same region as *qGY-6B* for yield (i.e., 100.50 cm on chromosome 6B). When the CR severity index was used for GWAS (instead of WH1 and WH2 alone), the same markers were identified but they showed a stronger association. A summary of the GWAS results including the QTL name, markers and their positions, marker-trait associations and their effects are presented in Table 2.

The QTLs discovered in this study were positioned on durum reference (Svevo) genome [41] along with QTLs reported in the literature (Figure 4D). The QTL regions on chromosome 6B reported in this study were positioned in close proximity to many previously reported QTLs [37,42,43,44,45,46,47,48] for root growth angle (RGA), Fusarium head blight (FHB), grain quality (yellow pigmentation, YP; yellow index, YI), thousand kernel weight (TKW) and yield (GY; Figure 4D).

### 2.6. The Combined Effect of qCR-6B and qSRA-6A on Yield

**Figure 5:**
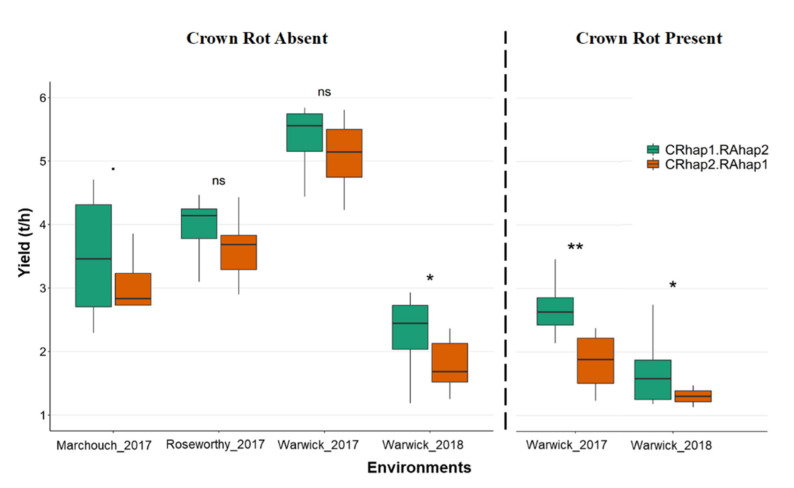
Comparison of genotypes that are segregating for root angle QTL (*qSRA-6A*) and CR QTL (*qCR-6B*): The genotypes were evaluated in the field across six environments (four in the absence of CR and two in the presence of CR). Green indicates the performance of genotypes that carry the resistant allele CRhap1 and wide root angle RAhap2. Orange indicates the performance of genotypes that carry the susceptible allele CRhap2 and narrow root angle allele RAhap1. Statistical tests were performed for haplotype groups within each environment, where the significance level is indicated as ** (*p* < 0.01) and * (*p* < 0.05). The mean yield benefit in the presence of CR was 1.1 t·h^−^^1^, whereas the mean yield benefit in the absence of CR across the four environments was 0.57 t·h^−^^1^.

Using the 2,541 high-quality DArTseq markers, we identified a set of genotypes that were closely related and segregated for both QTLs *qCR-6B* and *qSRA-6A.* This set was evaluated for their performance for yield under six different environmental conditions (Figure 5). In the presence of CR, lines that carried both favorable alleles for *qCR-6B* and *qSRA-6A* (referred to as CRhap1.RAhap2) significantly outperformed lines lacking both alleles (referred to as CRhap2.RAhap1). On average, the yield advantage was 0.90 t·ha^−1^ (*p* ≤ 0.01). In the absence of CR, lines carrying both favorable haplotypes (CRhap1.RAhap2) showed a trend for higher yield across the four environments (Figure 5). While within-environment differences were deemed statistically insignificant, the mean yield difference for lines carrying the favorable alleles was 0.57 t·ha^−1^ across all environments.

Overall, very large differences between average yield among trials in the absence of CR were noted, mainly driven by in-season rainfall as displayed in (Warwick_2017, Warwick_2018). The impact of CR was highlighted in Warwick_2018 when the yield losses were greater due to the combined effect of CR and low in-season rainfall. However, genotypes carrying CRhap1.RAhap2 were consistently superior to genotypes carrying CRhap2.RAhap1, maintaining higher yield in environments that varied for in-season rainfall (Table S1) and CR disease pressure.

## 3. Discussion

For the first time, we validated the value of root growth angle QTL *qSRA-6A* to support yield performance under rainfed conditions in Australia. In the absence of CR disease, lines carrying hap2 significantly out-yielded lines carrying hap1 by 0.57 t·ha^−1^. The results from wheat simulation studies report a yield increase of 50–60 kg·ha^−1^ for each additional millimeter of water accessed during the gain-filling period [49]. This could suggest that the yield advantage of 0.57 t·ha^−1^ observed in the current study likely arose due to improved access to approximately 10 mm of water in the soil profile. A recent study on durum wheat also reported a strong link between root architecture and yield in Moroccan drought environments, where deep root growth was associated with yield in dry environments [50]. However, in the current study, the *qSRA-6A-hap2* variant associated with wide root angle provided a yield advantage. This is not surprising because the value of different root systems is likely context dependent. For instance, in elite barley breeding lines, Robinson et al. [51] found that narrow root angle was advantageous in some environments but that, in others, a wide root angle was preferred. To add to the complexity, key genes in the flowering pathway, such as *Vernalization1*, are also known to influence root development [52]. Therefore, this could present challenges to breed varieties with a range of root configurations for all maturity classes required for different production systems.

In the presence of CR, *qSRA-6A* was not associated with yield (Figure 3D); however, lines carrying favorable alleles for both *qSRA-6A* and *qCR-6B* displayed significantly higher yield, where the average yield benefit was 0.9 t·h ^−1^ across the 2017 and 2018 yield trials (Figure 5). The inability to detect a yield effect associated with *qSRA-6A* alone was likely a result of the increased plot variability due to disease, as reflected by lower broad-sense heritabilities for most traits measured in the CR nursery. The *qCR-6B* QTL on chromosome 6B was mapped using the CR severity index (*qCR-6B*). The same interval was also mapped using individual readings for percentage of whiteheads (*qWH1-6B* and *qWH2-6B*) and yield in the presence of CR (*qGY-6B*). Interestingly, *qCR-6B* was also positioned in close proximity to previously reported QTLs for FHB resistance [44,46], which suggests that this could be a key genomic region for wheat responses to Fusarium in general.

In cereals, the stay-green phenotype can result from either conserving water early in the season or improving access to stored soil moisture late in the season [53,54]. This results in optimized water use to meet the demand during grain filling stage and maximized yield [35]. The QTL associated with stay-green traits were detected on chromosome 6B (*qSG-0.1-6B* and *qSG-integral-6B*), which were positioned 17.71 cm away from *qCR-6B*. Therefore, these loci appear to be independent. A moderate likelihood of recombining the two loci (i.e., 17.7% per generation) presents an opportunity for breeders to identify lines with various combinations of CR tolerance and stay-green traits. However, lines differing for the main haplotypes of *qCR-6B* also showed significantly different stay-green and root biomass phenotypes. Notably, this is not the first report of a link between root biomass and CR tolerance. A recent study in bread wheat found that genotypes with a higher total root biomass were more resistant to fungal infection [4]. This could be due to higher lignin/fiber content at the cellular level presenting a physical barrier for fungal growth and therefore providing enhanced resistance. Considering its association with stay-green and root biomass, *qCR-6B* could be involved in modulating canopy development early in the season and possibly root development to provide improved water-use strategies and access to water. These are likely the key mechanisms exploited by higher yielding durum lines, particularly in the presence of CR, because they lack genetic resistance per se.

## 4. Materials and Methods

### 4.1. Plant Material

A panel comprising 168 genotypes was evaluated in this study. The panel included 151 durum lines from the NAM population developed at the University of Queensland [39]. The NAM population was generated by crossing eight lines from ICARDA’s durum breeding program in Morocco with two Australian cultivars. The elite lines from ICARDA (Fastoz2, Fastoz3, Fastoz6, Fastoz7, Fastoz8, Fastoz10, Outrob4 and Fadda98) were used as founders and Australian cultivars (DBA Aurora, Jandaroi) were used as reference parents. The founder lines were selected due to their superior drought adaptation and have been used as parents in durum breeding programs targeting marginal rainfall regions of West Asia and North Africa. The reference parents are preferred by Australian growers for their high yield and quality parameters required by the pasta industry. The panel also comprised seven commercially released Australian durum varieties (Caparoi, Hyperno, Kalka, Saintly, Tjilkuri, WID 802, and Yawa).

### 4.2. Establishing a Crown Rot Field Screening Nursery

A CR field screening nursery was established at the Department of Agriculture and Fisheries Queensland (DAFQ), Hermitage Research Facility (28^o^12′40″ S; 152^o^06′06″ E), Warwick, Queensland. This nursery was prepared for over three years (2014–2016) and was made available for CR screening in 2017. A pure culture of *F. pseudograminearum* isolates was isolated from infected wheat stubble collected from grower fields in Brookstead, Queensland, as described by Alahmad et al. [40]. The pure culture was used to inoculate freshly ground millet, mixed with distilled water and placed inside sealed plastic bags. The bags were placed at 25 °C for 6 weeks to accommodate mycelium growth. The colonized ground millet was then air-dried, mixed with the bulk seed of a very susceptible Australian durum wheat variety (Jandaroi) and planted in the nursery for initiating disease infection during germination. Throughout the late grain-filling stage, CR symptoms were evident on the base of the plants and the stems, resulting in the formation of whiteheads. At maturity, Jandaroi was ploughed into the soil and stubble was retained to build up inoculum levels in the soil. This process was repeated six times during the period spanning 2014–2016 to achieve high levels of inoculum and significant CR disease pressure in the screening nursery.

### 4.3. Field Experiments

#### 4.3.1. Yield Trials in the Presence of CR

The panel of durum lines was evaluated for yield under high disease pressure in the CR screening nursery in 2017 and 2018. In both years, mini yield plots were sown using a partial replication (p-rep) design where 50% of genotypes were replicated in a row-column grid to maximize the number of tested genotypes, as described by Cullis et al. [55]. Plots contained 4 rows and were 4 meters in length (i.e., 4 m^2^).

In 2017, the panel was subjected to extensive phenotyping for a number of traits, including days to flowering (Zadock 65), plant height (PH; cm), weekly measures of normalized difference vegetation index (NDVI) recorded from flowering time until physiological maturity (Zadock 93) and CR symptoms (scores 0–9 and % of whitehead). NDVI was captured using a handheld GreenSeeker™ (NTech Industries Inc., Ukiah, CA, USA) following procedures described by Lopes and Reynolds [56]. Raw NDVI measures recorded over time for each plot were used to model stay-green traits [31], including onset of leaf senescence (OnS), mid-point of leaf senescence (MidS), near completion of leaf senescence (EndS) and stay-green integral (SGint), as displayed in Table 3. To phenotype CR severity, all plots were scored for the percentage of whiteheads due to CR infection at four (WH1 %) and two weeks (WH2; %) before physiological maturity. Further, to quantify CR symptoms displayed as stem browning, 10 plants per plot were manually extracted from the inner two rows and scored for stem browning discoloration using a 0–9 scale, where 0 is fully resistant and 9 is the most susceptible [40]. A weighted index for CR severity was calculated by combining the datasets derived from the percentage of whiteheads and the stem browning discoloration scores, where equal weighting was applied to each of the three measures (WH1, WH2 and stem browning scores). At crop maturity, plots were harvested using a mechanical plot harvester and yield was recorded.

To validate associations between trait QTL and yield under CR disease pressure, the panel was evaluated for yield in the CR screening nursery in 2018.

#### 4.3.2. Yield Trials in the Absence of CR

Yield trials were performed in the absence of CR under rainfed conditions at Warwick (Queensland, Australia) in 2017 and 2018. Importantly, the trials were conducted on a block of land that had very low levels of CR inoculum that was insufficient to cause visible disease symptoms for very susceptible durum varieties. The block was also located in close proximity to the CR nursery block (only 200 m away). The yield trials were also sown on the same day as the respective CR experiments and, therefore, were exposed to similar water stress throughout the season. The trials also used the same mini-plot and p-rep design, as described above.

In 2017, the panel was subjected to extensive phenotyping for a number of traits, including flowering date, PH and weekly NDVI measurements from flowering time until physiological maturity (to calculate stay-green traits, as detailed above).

To further investigate trait QTL associations with yield across a broader range of environments, the panel was subjected to yield trials at three additional sites. In 2017, the panel was evaluated at Roseworthy (34°30′08.5″ S; 138°41′30.2″ E), South Australia and Marchouch (33°36′48.0″ N; 6°43′04.8″ W), Morocco, and in 2018, the panel was yield tested at Warwick, Queensland. All three trials were conducted in the absence of disease, and no visible CR symptoms were observed. Site management for all trials, including chemical control for weeds and fertilizer for maximizing crop productivity, was conducted on a needs basis and by using the industry standard best practice. Additional site information is provided in Table S1.

### 4.4. Phenotyping Root Biomass under Controlled Conditions

To investigate the relationship between root biomass and yield under CR conditions, a subset of the panel (40 genotypes) was selected according to the haplotype at the major QTL for root angle *qSRA-6A* (i.e., hap1 = narrow, *n* = 20; and hap2 = wide, *n* = 20) [39]. The 40 genotypes were evaluated for root biomass using the method reported by Voss-Fels et al. [57] with slight modifications. Here, ANOVApot^®^ pots (137 mm diameter and 140 mm height) were filled with 1700 g of sand (with particle size ranging from 0.075–4.75 mm) to facilitate efficient cleaning of roots. A randomized complete block design (RCBD) was adopted, with four plants per genotype in each 1.40 L pot and three replicates. Fifteen pots were placed in a container fitted with capillary matting to enable water and nutrient flow through the bottom of the pots. Hydroponic solution was added to each container (1.50 mL of Cultiplex per litre of de-ionised water) with the solution reaching the base of the pots and the solution level maintained throughout the experiment. The concentration of the solution was optimized according to the plant growth stage as follows: 1–10 d: 1.50 mL/L, 11–17 d: 2 mL/L, 18–22 d: 2.50 mL/L and 23–28 d: 3 mL/L.

The seeds were imbibed and subjected to cold treatment (5 °C) for three days to synchronize germination across genotypes, and the germinated seeds were sown under diurnal glasshouse conditions with 22 °C day/17 °C night temperature. At the early tillering stage (Zadock 22), plants were extracted with minimal disruption to the roots by placing the pot in a bucket of water and by washing off the sand in clean water. The roots and shoots of the four plants were separated, and the roots were placed in a dehydrator at 65 °C for 72 h. After drying, root biomass was weighed using a balance with 0.0001 g accuracy (AND, HR–200, A&D Company Limited, Tokyo, Japan). The measurements of root biomass for the four plants were considered as a single experimental unit in the analysis.

### 4.5. Analysis of Phenotype Data

The panel of 168 genotypes tested in the six field trials (two in the presence of CR and four in the absence of CR) were phenotyped for yield and other traits, which were analyzed using the *ASReml–R* package [58] in R software *V3.4.3* [59]. To account for spatial variation in the field sites, a mixed linear model was fitted in *ASReml–R*. In this model, genotype was fitted as a fixed effect while replicates and the field grid of row and column were fitted as random terms. Best linear unbiased estimates (BLUEs) were calculated and used as adjusted mean values for each trait for each genotype evaluated in each environment. The broad-sense heritability (H^2^) and coefficient of variation (CV) was also calculated for all traits.

To investigate the relationship between yield and traits (stay-green traits, PH and days to flowering), the Pearson’s correlation coefficient (*r*) was calculated using the mean values (BLUEs) for each genotype.

### 4.6. Genome-Wide Association Mapping

The 168 durum lines were previously genotyped, as reported by Alahmad et al. [40]. Briefly, a total of 2541 high-quality genome-wide markers were used to investigate genomic regions associated with CR severity traits, stay-green traits and yield. The mean marker interval was 81.8 Mbp, which varied across the 14 chromosomes: 218.2 for 1A, 131.7 for 1B, 62.3 for 2A, 37.1 for 2B, 82.8 for 3A, 71.9 for 3B, 101.7 for 4A, 70.5 for 4B, 121 for 5A, 63.7 for 5B, 75.6 for 6A, 46.6 for 6B, 78.4 for 7A and 72.9 for 7B. The BLUEs for each trait were used in a mixed model implemented in the R package *GenABEL* [60]. The marker-trait associations were calculated using a two-step mixed linear model approach that increases detection power without increasing the empirical type I error [61]. The model was adjusted for population stratification by including identity-by-state estimates for genotype pairs (as a kinship matrix) and a principal component adjustment that uses the first four principal components as fixed covariates to account for variation due to population structure. For the identification of significant marker-trait associations and to control the probability of false positives, an arbitrary threshold of −log_10_(*P*) ≥ 3 was applied.

To determine the main haplotypes associated with the key QTL for CR tolerance, the local LD was calculated for the significant markers in the region. Markers with pairwise *r^2^* values ≥ 0.7 were subjected to haplotype analysis, resulting in two major haplotype variants (*n* ≥ 27). Furthermore, the allelic effect of the CR and SRA (*qSRA-6A*) QTLs on stay-green and yield was investigated by comparing the performance of genotypes that were segregated for the QTL. Genotypes that carried both favorable alleles for SRA and CR QTL were compared to genotypes that lacked both alleles using Tukey’s HSD (honestly significant difference) test with a family-wise error rate of 5%.

### 4.7. Alignment of QTL for Aboveground Traits Across Studies

The QTLs identified in this study were aligned with genomic regions previously reported in the literature for key drought-related traits; pathogen susceptibility, yield as well as grain quality [42,43,44,45,46] were positioned on the Svevo durum physical map [41] using MapChart V2.3 [62].

## 5. Conclusions

This research investigated the value of above- and belowground adaptive traits to improve durum yield in rainfed production systems with and without CR disease. This study demonstrated that traits related to water-use efficiency and access to water, such as stay-green and root system architecture, can improve crop performance and can reduce losses due to drought and CR stress. Interestingly, the 6B QTL region (100.50–101.26 cm) initially mapped for tolerance to CR and yield was also associated with stay-green in the presence of CR and increased root biomass under controlled conditions. This suggests that genes underlying the QTL could modulate the size of the canopy and/or root system size to improve water-use efficiency and could reduce the impact of CR disease. The 6B locus appears to be independent to the major root angle locus *qSRA-6A* [39], which highlights the opportunity to combine traits that optimize canopy and root system architecture to enhance the performance of durum crops under abiotic and biotic constraints.

## Figures and Tables

**Table 1:** Trait minimum (Min), maximum (Max), adjusted mean response, broad sense heritability (H^2^) and coefficient of variation (CV%) for 168 genotypes evaluated in the presence and absence of crown rot in 2017 Warwick, Queensland.

Traits	Crown Rot Present					Crown Rot Absent				
	Min	Max	Adjusted Mean	H^2^	CV %	Min	Max	Adjusted Mean	H^2^	CV %
Yield (GY; t·h^−1^)	0.58	3.94	2.30	0.67	27.0	3.5	6.4	4.9	0.68	13.4
Plant height (PH; cm)	52.9	95.8	66.7	0.45	8.7	48.0	85.5	63.4	0.79	8.9
Time to flowering (DTF; d)	99.7	112.4	106.8	0.18	2.0	96.6	106.4	101.1	0.31	2.2
Thousand kernel weight (TKW; g)	28.3	48.7	37.7	0.77	11.2	32.5	53.8	41.9	0.72	11.0
Onset of leaf senescence (OnS; d)	7.0	19.0	6.5	0.41	13.4	4.7	26.9	16.7	0.54	11.3
Mid-point of leaf senescence (MidS; d)	5.3	26.9	16.8	0.48	26.9	15.6	31.8	25.3	0.45	13.4
Near completion of leaf senescence (EndS; d)	19.2	47.7	30.0	0.25	16.5	29.2	43.3	36.3	0.26	8.3
Stay-green integral (SGint)	16.4	29.8	23.0	0.5	11.0	20.4	30.8	24.9	0.47	9.9
CR severity (CR)	2.3	8.8	5.9	0.24	19.2	NA	NA	NA	NA	NA
1st reading whiteheads (% WH1)	1.1	65.2	21.3	0.45	65.4	NA	NA	NA	NA	NA
2nd reading whiteheads (% WH2)	1.9	77.2	37.1	0.57	48.0	NA	NA	NA	NA	NA

**Table 2:** Summary of QTLs detected in the panel of 168 genotypes evaluated in the presence and absence of crown rot (CR) at Warwick in 2017.

Trait ^a^	QTL Name	Marker	Chromosome	cm ^b^	−log_10_(*P*) ^c^	Marker Effect ^d^
Plant height	*qPH-6B*	995614	6B	2.19	3.42	5.339
		1008368	6B	2.19	3.37	5.305
EndS	*qSG-* *0.1-* *6B*	3025934	6B	82.79	3.13	1.611
SGint	*qSG-* *integral-* *6B*	3025934	6B	82.79	3.01	0.750
Yield (t·h^−1^)	*qGY-6B*	1023342	6B	100.50	3.26	−0.215
		2254131	6B	100.50	3.12	−0.203
		4992135	6B	100.50	3.15	−0.203
WH1	*qWH1-6B*	1023342	6B	100.50	3.88	7.350
		2254131	6B	100.50	3.44	6.744
		4992135	6B	100.50	3.65	7.025
		1039837	6B	101.26	3.41	6.800
		2256390	6B	101.26	3.60	6.998
WH2	*qWH2-6B*	1023342	6B	100.50	3.40	8.582
		2254131	6B	100.50	3.36	8.386
		4992135	6B	100.50	3.58	8.777
CR severity	*qCR-6B*	4992135	6B	100.13	4.14	−0.590
		1023342	6B	100.50	4.20	−0.598
		2254131	6B	100.50	3.93	−0.568
		2256390	6B	101.26	3.25	−0.524
		1039837	6B	101.26	3.10	−0.508

^a^ Plant height measured in centimeters (cm); EndS is the number of days from flowering time to 90% senescence; SGint is the area under the curve of the stay-green model from flowering time to full senescence; t·h^−1^ is tons per hectare; WH1 and WH2 are the percentages of whiteheads due to CR infection within each plot collected at 4 and 2 weeks before physiological maturity, respectively; and CR severity is the crown rot severity index calculated using the two readings for percentage of whiteheads and stem browning. ^b^ Chromosomal positions based on the Svevo durum wheat map. ^c^ −log10(*P*), where a threshold of *p* < 0.001 was applied for significant marker-trait associations. ^d^ Positive or negative associations between different traits based on DArTseq alleles at each locus.

**Table 3:** Stay-green traits adopted from Christopher et al. [30] based on a fitted curve to the periodic normalized difference vegetation index (NDVI) measurement (number of days) collected from flowering time until full canopy senescence.

Abbreviation	Stay-Green Trait	Description
OnS	Onset of leaf senescence	Number of days from flowering to 90% of greenness
MidS	Mid-point of leaf senescence	Number of days from flowering to 50% of greenness
EndS	Near completion of leaf senescence	Number of days from flowering to 10% of greenness
SGint	Stay-green integral (senescence integral)	Total stay-green parameter referring to the green leaf area duration from flowering to full senescence

## Supplementary Materials

Supplementary materials can be found at https://www.mdpi.com/1422-0067/21/15/5260/s1.
